# Gut Microbial Dysbiosis and Implications in Solid Organ Transplantation

**DOI:** 10.3390/biomedicines12122792

**Published:** 2024-12-09

**Authors:** Cathlyn K. Medina, Berk Aykut

**Affiliations:** 1 Duke University School of Medicine, Durham, NC 27710, USA; 2 Department of Surgery, Duke University, Durham, NC 27710, USA

**Keywords:** gut microbiome, microbial dysbiosis, solid organ transplant, outcomes

## Abstract

The gut microbiome has been shown to play a significant role in solid organ transplantation, potentially influencing graft function and patient outcomes. Dysbiosis, characterized by reduced microbial diversity and an increase in pathogenic taxa, has been linked to higher incidences of allograft rejection, graft dysfunction, and post-transplant mortality. Several studies suggest that the gut microbiome might be able to serve as both a biomarker and a therapeutic target, potentially guiding personalized immunosuppressive therapies and other interventions to improve outcomes after solid organ transplantation. As summarized in this review, clinical studies have shown that specific microbial shifts correlate with adverse outcomes, including acute rejection and chronic allograft dysfunction. As research surrounding the relationship between the gut microbiome and solid organ transplant progresses, the integration of microbial analysis into clinical practice has the potential to revolutionize post-transplant care, offering new avenues to improve graft survival and patient quality of life. This review aims to provide a comprehensive overview of the relationship between gut microbial dysbiosis and transplantation outcomes, emphasizing the impact on kidney, liver, lung, and heart transplant recipients.

## 1. Introduction

Although the complex interplay between the human gut microbiome and the development and modulation of illness and disease states has been widely recognized [1], causal modulatory pathways and host-microbe interactions still remain poorly understood. Microbial dysbiosis, characterized by decreased microbial diversity with a corresponding rise in pathogenic microbes, has recently gained attention as a primary source of host pathological inflammatory responses. As such, various studies have outlined a definitive association between gut microbial dysbiosis and gastrointestinal pathologies [2,3,4]. For example, alterations in microbial taxa and a reduction in overall diversity of the intestinal microflora are characteristic for patients with inflammatory bowel disease (IBD) [2], along with the generation of IBD-specific mucosal biofilms which may trigger pro-inflammatory responses within the local host tissues [2,3,4,5]. As further support, the introduction of probiotics in a murine model of ulcerative colitis demonstrated recovery of beneficial microbiota and a reduction in inflammatory cytokines, indicating an improvement in the disease state [6]. In addition to direct enteric effects, microbial dysbiosis has also been implicated in several extraintestinal pathologies, including cancer progression and resistance to various immunotherapies [7,8,9,10]. While many of the principles governing the interplay between the gut microbiome and systemic immunity remain to be discovered, direct and indirect communication via metabolites between the enteric microflora and the host immune system have been well-described [11,12]. As such, therapeutic targeting of the gut microbiome has shown promise as an avenue of possible intervention and has become an area of active investigation over the last few decades in many different disease states, including solid organ transplantation.

In patients with end-stage organ failure, referred to from here on out as end-stage renal disease (ESRD), end-stage liver disease (ESLvD), end-stage lung disease (ESLgD), and advanced heart failure (AHF), transplantation remains the only life-saving treatment currently available. Despite significant medical and surgical advances, transplant-associated morbidity (infection, rejection, graft failure) and mortality remain major challenges [13,14]. As a possible avenue of mitigation for these significant complications in the field of allotransplantation, an enhanced understanding of the nuanced interactions between the gut microbiome and systemic immunity of transplant recipients could serve to guide potential therapeutic interventions. The complex microenvironment of the human gut is characterized by a diverse and variegated ecosystem encompassing up to 100 trillion cells that span bacteria, viruses, protozoa, and fungi [15]. In the human gut, greater than 50 unique phyla have been reported, with *Actinobacteria*, *Bacteroidetes*, *Firmicutes*, *Proteobacteria*, *Tenericutes*, and *Verrucomicrobia* comprising a significant portion of the dominate phyla [16]. Notably, the gut microbial communities of transplant recipients significantly differ from those of the healthy individuals and are characterized by decreased microbial diversity, increased viral infections, and colonization with drug-resistant bacteria [17,18,19,20]. Further compounding these existing perturbations in the enteric microbial community, immunosuppressive medications required by allograft recipients compromise the host immune system, diminishing the recipient’s ability to regulate the bacterial microenvironment potentially contributing to the exacerbation of the dysbiotic state.

**Figure 1:**
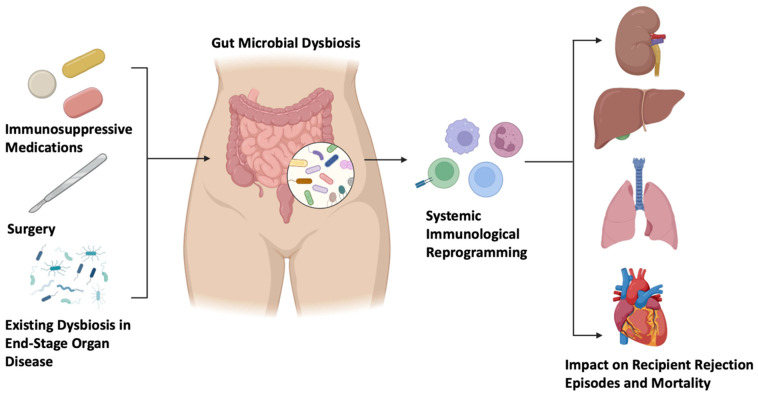
Schema summarizing the impacts of immunosuppressive medications, surgery, and existing dysbiosis in end-stage organ disease on the gut microbiome and the subsequent influence on the recipient immune system and resulting episodes of allograft rejection or mortality. Images created using Biorender.

Several pre-clinical studies in murine models have demonstrated the immune modulatory effect of the gut microbiome on alloimmunity and rejection, highlighting the direct interplay between the gut microbiome and systemic immunity and its potential for therapeutic targeting in solid organ transplantation [21,22]. Clinical studies provide further evidence for a link between gut microbial dysbiosis and adverse outcomes in solid organ transplant recipients [23,24]. With the growing evidence of the influence of enteric microbes on post-transplant outcomes, we herein provide a comprehensive overview of the relationship between the intestinal microbiome, allograft rejection, and mortality in human kidney, liver, lung, and heart transplant recipients (Figure 1).

## 2. The Gut and the Immune System

While specifics surrounding the complex interactions between the gut microbiome and systemic immunity remain to be uncovered, recent findings shed light on how these interactions may unfold [12]. Under normal physiologic conditions, metabolites from enteric microbes both directly and indirectly affect host immunity and modulate systemic immune responses [11,12]. Common bacterial components of the gut microbiome include *Faecalibacterium prausnitzii*, *Roseburia intestinalis*, and *Anaerostipes butyraticus* [25]. These symbiotic genera digest carbohydrates via fermentation, a byproduct of which are short-chain fatty acids (SCFAs) [26,27], which are used locally by colonocytes and systemically by other tissues as a source of fuel and aid in the regulation of host immune cells [28,29]. SCFAs are enriched by the gut lumen and consist primarily of butyrate, propionate, and acetate [12,28]. They affect intestinal immune function via direct action on leukocytes and intestinal epithelial cells (IECs), thus playing an integral role in maintaining intestinal barrier integrity and mitigating local inflammatory processes [30,31,32].

Identification and uptake of SCFAs by IECs aids in developing the host immune response and subsequently impacts how the local enteric environment is regulated and preserved. The maintenance of a homeostatic environment is a complex task that requires identification of invasive pathogens and selective tolerance to symbiotic and commensal microbiota. Guided by the host microbiome, mucosal and systemic immune responses are regulated by various T_reg_ cell subsets [33,34,35,36]. Thus, maintenance of the gut microbiome is vital in maintaining host immunologic homeostasis [37,38]. As such, disruptions in gut bacterial composition and functionality can have deleterious effects on host health [39]. For example, *Bifidobacterium*, a commensal bacteria, reduces intestinal pH via SCFA production which in turn prevents the growth of opportunistic bacteria like *Escherichia coli* [30]. In addition to creating an unfavorable environment for the survival of invasive pathogens, commensal microbes also reduce the availability of vital nutrients for aerobic pathogens. Notably, the *Enterobacteriaceae* genus are facultative anaerobes and consume any residual oxygen that virulent microbes, such as *Shigella flexneri*, require to secrete their toxins into the intestinal lumen [40].

Overall, gut microbial dysbiosis has been implicated in an array of illnesses and disease states [39,41] and can be linked to a plethora of etiologies including host diet, mucosal damage, host genetic factors, or the administration of antibiotics or immunosuppressive medications [42,43]. Among transplant allograft recipients, immunosuppressive therapy leads to persistent and longitudinal effects on the intestinal microflora through various ways which have yet to be fully characterized.

## 3. Immunosuppressive Therapy and the Intestinal Microbiome

Among solid organ transplants recipients or patients suffering from autoimmune diseases, immunosuppressive medications are often a life-long requirement. Most immunosuppressive regimens for organ transplant recipients include variations of corticosteroids, calcineurin inhibitors, and antimetabolites [44]. These treatments are commonly accompanied by various side effects including infections, hypertension, diabetes, and gastrointestinal upset. In addition to these secondary effects, it is well documented that solid organ transplant recipients have accompanying alterations in their gut microbiome [45,46]. While the initially profound gut microbial dysbiosis may improve over time, persistent deviations that can potentially affect long-term outcomes of allograft recipients often remain [47,48].

### 3.1. Corticosteroids

The use of corticosteroids, most commonly dexamethasone, is one of the cornerstones of the immunosuppressive treatment that is used in solid organ transplant recipients to prevent allograft rejection. To study the short-term effects of corticosteroids on the gut microbiome in a controlled environment, Zhao et al. utilized a murine model and reported a decrease in gut microbial richness and diversity as quantified by the Shannon diversity index, an alpha-diversity measurement of richness and evenness [49]. Prior studies have demonstrated that the use of corticosteroids results in alternations of the relative abundance of microbes, particularly *Bacteroidetes* and *Firmicutes*, at a phylum level, in comparison to controls in both clinical and pre-clinical models [49,50,51,52,53]. In another study, Zimmermann et al. closely investigated the ability of the different species of human enteric microbiota to metabolize different oral medications and demonstrated that most drugs undergo chemical alteration by the gut microflora [54]. This work sheds light on how *Clostridium scindens* and *Propionimicrobium lymphophilum* facilitate the transformation of glucocorticoids to androgens within the gastrointestinal tract. As these bacteria enable the conversion of steroids to androgens, the resulting reduction in levels of glucocorticoids leads to diminished immunosuppressive effects and increased recipient vulnerability to allograft rejection or the negative secondary effects of persistently elevated androgen levels [54,55]. In addition to alterations in the relative abundance of existing flora and the direct effects on drug metabolism, studies have demonstrated that glucocorticoids also alter the integrity of the intestinal barrier and its ability to regulate local bacterial proliferation, potentially further impacting host drug metabolism [53,56]. While the mechanisms behind these observations remain not fully understood, the link between the gut microbiome and the resulting impact on both local and systemic processes is also evident with other immunosuppressive medications.

### 3.2. Tacrolimus

Tacrolimus, another commonly used immunosuppressive medication, is similarly associated with changes in the gut microbiome. Tacrolimus binds to the FK506-binding protein and the resulting complex specifically binds to and inhibits calcineurin phosphatase, causing a decrease in inflammatory cytokines [57]. Several studies have demonstrated alternations in the gut microbiome after treatment with tacrolimus, although relative abundancies and compositions varied between studies [51,58,59,60,61]. In a recent study, Degraeve et al. uncovered that alterations in gut microbiota may partly contribute to the variability in tacrolimus trough levels through effects on ABCB1, an efflux transporter that is more abundantly expressed in patients with persistently reduced tacrolimus levels [61]. In addition to changes in microbial richness, particular bacteria have been found to directly impact the metabolism of tacrolimus. *Faecalibacterium prausnizii* and *Clostridiales* transform tacrolimus into a less active compound, “M1”, a metabolite which has been found in the blood of kidney transplant recipients, leading to a possible increased risk of graft rejection [62,63]. Interestingly, not only does the administration of tacrolimus affect the gut microbiome, but there is also preliminary evidence that resulting alterations may be dose-dependent. Jiang et al. demonstrated that in rat liver transplant recipients, intermediate doses of tacrolimus were associated with increased intestinal microbial richness and a decrease in potentially harmful bacteria [64]. Conversely, the extremes of higher and lower doses of tacrolimus were associated with worse liver allograft outcomes [64]. Further investigation into the exact mechanisms through which concentrations of tacrolimus are impacted by the local microenvironment are needed, however, this preliminary evidence suggests that there may be significant benefit to determining optimal trough levels or potential targeted microbial repopulation to optimize recipient outcomes.

### 3.3. Mycophenolate Mofetil

In addition to tacrolimus, another commonly used drug among transplant recipients is mycophenolate mofetil (MMF). MMF, a prodrug of mycophenolic acid (MPA), is a non-competitive, selective inhibitor of inosine monophosphate dehydrogenase, an enzyme required for purine synthesis [65]. Among renal transplant recipients taking MMF, significant gastrointestinal side effects have been demonstrated, leading to digestive disorders in up to 50% of patients [66]. Moreover, mice undergoing treatment with the prodrug have been found to have significant gut microbial dysbiosis [67] with decreased biodiversity and increased *Firmicutes/Bacteroidetes* ratios along increased abundances of opportunistic pathogens [68], providing further evidence for the intestinal effects of MMF. While the exact mechanisms of gut microbial dysbiosis have still yet to be revealed, MMF is commonly associated with the development of colitis, secondary to an enhanced relative abundance of MPA, a colitogenic substrate [67]. In addition to differences in relative abundances of microbes, it has also been reported that MMF requirements can vary between different types of transplant recipients. In a recent study, Khan et al. demonstrated that differences in B-glucuronidase activity between kidney transplant and hematopoietic stem cell transplant recipients could in part explain the different dose requirements between these patient populations [69]. Taken together, these works demonstrate the intricate interplays between the microbiome and metabolism of immunosuppressive drugs; however, further studies are needed to uncover how to best optimize these interactions to advance outcomes after solid organ transplantation.

## 4. The Gut Microbiome and Outcomes After Kidney Transplantation

### 4.1. Gut Dysbiosis in Patients with End Stage Renal Disease

Alterations in the profile of the gut microbiome have been well-documented in ESRD patients, providing evidence for this patient population’s significant dysbiosis, which often develops prior to even receiving a renal transplant [23,70,71,72,73,74]. ESRD-specific alterations may be secondary to an array of different processes and include increases in relative abundances of *Escherichia*, *Clostridium*, *Streptococcus*, *Methanobrevibacter smithii*, and *Ruminococcus torques*, a bacteria associated with disruption of the gut barrier [75]. Additional work by Sampaio-Maia et al. highlights an association between ESRD and decreased enteric abundances of *Bifidobacteriaceae*, *Lactobacillaceae*, *Bacteroidaceae*, and *Prevotellaceae*, and, conversely, increased levels of *Enterobacteriaceae* [72]. This higher abundance in Proteobacteria phyla in comparison to healthy individuals characterizes the intestinal flora of ESRD patients and is thought to be a marker of disease. Early intervention on this existing dysbiosis after solid organ transplant may help to minimize future kidney allograft complications.

### 4.2. Gut Dysbiosis After Transplantation and Renal Allograft Rejection

Some of the most feared complications after renal transplantation include impaired allograft function or rejection secondary to T-cell-mediated rejection and/or anti-body mediated rejection [76]. Previous work has demonstrated that alterations in the recipient intestinal microflora may influence allograft function and rejection and could be a potential target to help mitigate graft survival and life expectancy in kidney transplant recipients. To further elucidate the influence of the microbiome on renal allograft outcomes, Kim et al. sought to investigate if genetic distance between gut microbiota profiles was predictive of renal allograft function and outcomes. In their study, the authors identified differences in gut microbiota as a key predictor of allograft outcomes, with microbial distance showing excellent accuracy in predicting eGFR at 6 months, holding promise as an even better predictor than human leukocyte antigen incompatibility [74].

Changes in the gut microbiota secondary to kidney transplantation have been reported in previous studies and remain an active area of research in both the short and long term to determine the effect on allograft outcomes (Table 1). Early analysis of the gut microbiome pre- and post- renal transplantation by Lee et al. demonstrated increased quantities of Proteobacteria, a pathogenic phyla, and a decrease in *Bacteroides*, a commensal genus [77]. As previously detailed, gut microbes, such as *Clostridia*, *Bacteroides fragilis*, and *Bacteroides thetaiotaomicron*, directly influence the development of immune tolerance through differentiation of cell types and cytokine production [78,79,80]. Notably, *Bacteroides* species have well-described immunomodulatory functions, particularly affecting T_reg_ cells, which play a key role in mediating tolerance the severity of allograft rejection [81,82,83]. Longitudinal analysis of the recipient microbiomes after transplantation by Swarte et al. demonstrated initial maintenance of the same dysbiotic state existing prior to transplantation, potentially as a result of the new requirement for immunosuppressive medications [23,84,85]. Overall, there remained a significant decrease in microbial diversity from weeks to 20 years after transplantation [23,85].

Work by Guirong et al. revealed an elevated relative abundance of the microbes *Bacteroides* and *Enterobacteriaceae* among renal transplant recipients in comparison to their healthy counterparts [89]. This increase in *Enterobacteriaceae* uncovered by the authors was distinctive from patients with ESRD undergoing hemodialysis, suggesting that an increase in *Enterobacteriaceae* species may be unique to renal transplant recipients. Conversely, Chen et al. uncovered an interesting decrease in the presence of *Bacteroides*, *Megamonas*, and *Prevotella* in ESRD patients undergoing hemodialysis [90]. Although these findings conflict with the previous results, this discrepancy may be attributed to the fact that the control population used by Chen et al. consisted of patients undergoing hemodialysis, which is known to inherently alter the gut microbiome [91]. Additionally, there were decreased abundancies of *Ruminococcaceae*, *Lachnospira*, and *Faecalibacterium* after transplantation, all SCFA-producing bacteria which, by the mechanisms described previously, are vital for immune regulation. In contrast to other studies, a recent study by Chan et al. demonstrated an increased abundance of the SCFA-producing bacteria, *Roseburia intestinalis* and *F. prausnitzii*, following transplantation [92]. The non-uniform control populations amongst the different studies could potentially explain these ostensibly contrasting results, as the authors compared pre-transplant samples from the recipients rather than utilizing a baseline of the general population. Similarly, six years after transplant, there is a relative decrease in other butyrate-producing bacteria, including *Eubacterium rectale* and *Roseburia* [18]. Additional work by Souai et al. provided additional insight into the longitudinal changes in gut flora up to 22 years after renal transplantation [93]. This analysis provided further evidence for the chronic lack of microbial diversity and richness after transplantation with a persistent increase in Proteobacteria over time, consistent with the previously discussed studies. The authors highlighted that the early post-transplant gut microbiome, was initially characterized by an abundance of *Asteroleplasma*, *Roseburia*, *Faecalibacterium*, and *Bacteroides*, which then transitioned to *Rikenellaceae RC9*, *Dialister*, *Parabacteroides*, *Sutterella*, *Escherichia*/*Shigella*, and *Succinivibrio* over the remainder study period [93].

Furthermore, Wang et al. reported lower abundancies of *Clostridia*, *Clostridiales*, and *Roseburia* accompanied by a higher abundance of *Erysipelotrichi*, *Erysipelotrichacea*, *Lactobacillales*, and *Bacilli* [87] among recipients with acute antibody-mediated rejection. Exclusion criteria included recipients with any recent infection or medical intervention to isolate and strengthen any associations between microbes and the development of graft rejection; however, any altering effects secondary to immunosuppressive medications could not be entirely ruled out. Similarly, an association between proinflammatory taxa and renal allograft rejection has been shown by Visconti et al., who demonstrated an increased quantity of *Escherichia-Shigella*, *Ruminococcus gnavus*, and *Anaerotruncus* in comparison to controls [86]. Studies by Lee et al. provide further evidence for a link between gut microbial perturbations and the development of acute rejection. In patients developing acute transplant rejection within 3 months of transplant, relative abundances of *Clostridiales*, *Bacteroidales, Eubacterium dolichum*, and *Ruminococcus* were decreased while those among *Lactobacillales*, *Enterococcus*, *Anaerofilum*, and *Clostridium tertium* were increased in comparison to recipients that did not develop acute rejection [77]. Despite these differences in microflora, those experiencing rejection also received antibiotics during the study period, making it challenging to isolate a causal connection between specific bacterial taxa and renal allograft rejection. Lastly, although constrained by a small sample size, Yan et al. demonstrated a significantly increased abundance of *Curtobacterium* in kidney transplant recipients experiencing borderline change for acute T-cell mediated rejection compared to recipients without [88].

While not specific to particular microbes, a recent study by Carron et al. assessed outcomes of gut bacterial translocation on rejection in kidney transplant recipients. Interestingly, the authors found that elevated inflammatory markers in ESRD patients were protective against acute rejection in allograft recipients. Additionally, while concentrations of LPS, indicative of gut bacterial translocation, remained stable after renal transplantation, LPS activity decreased. The authors hypothesized that this may be due to chronic stimulation of myeloid cells by bacterial products generating endotoxin tolerance [94,95], resulting in dampening of immune responses and subsequent attenuation of acute rejection in kidney transplant recipients [96].

### 4.3. Gut Dysbiosis and Post-Transplant Mortality in Renal Allograft Recipients

Several studies have demonstrated that alterations in the recipient gut microbiome can result in increased recipient mortality in hematopoietic stem cell transplant recipients [97]. Interestingly, when the association between alpha diversity of the gut microbiome and overall renal transplant recipient survival was assessed by Swarte et al., there was no significant correlation [23]. However, assessment of the Aitchison distance between the enteric flora of renal transplant recipients and the general population demonstrated a positive association between Aitchison distance and likelihood of death, with a unit increase in Aitchison distance correlating with a 69% increase in mortality risk [23]. Prior studies by Salosensaari et al. and Peled et al. have demonstrated previous specific microbial markers associated with increased risk of host mortality in the general population and hematopoietic cell transplant recipients, respectively [97,98]. Interestingly, renal transplant recipients did not display any significant interactions between the local enteric microenvironment and mortality after kidney transplant [23].

## 5. The Gut Microbiome and Outcomes After Liver Transplantation

### 5.1. Gut Dysbiosis in Patients with End Stage Liver Disease

Similar to patients with ESRD, those with ESLvD experience alterations to their gut microbiome associated with their disease process [99]. In their recent study, Swarte et al. demonstrated that ESLvD patients had the largest genetic distance from the general population and a correspondingly lower microbial diversity [23]. Parsing out indications for transplant as cirrhosis vs acute etiology, patients with cirrhosis were found to have a significantly higher Shannon diversity index, overall demonstrating a state of baseline dysbiosis in the ESLvD population and shift away from a healthy gut microenvironment. Further breakdown of the microbial species present in patients with ESLvD demonstrated a significant increase in the genera of *Escherichia*, *Clostridium*, and *Streptococcus* [23,99]. Alterations of the gut barrier in patients with cirrhosis [100] has been associated with the development of systemic inflammatory responses and subsequent complications of this disease state among cirrhotic patients [101,102]. Prior studies performed by other investigators demonstrated associations between the post-transplantation gut microbiome and corresponding disease severity and complications [17,103]. While it is possible that transplantation could in isolation reinstate the gut-liver axis, the surgical intervention alone plus the numerous additional insults that are associated with liver transplantation continue to contribute to the deterioration of the gut barrier and can lead to significant morbidity and mortality among liver transplant recipients [104].

### 5.2. Gut Dysbiosis After Transplantation and Liver Allograft Rejection

Ponziani et al. demonstrated that compared to healthy controls, liver transplant recipients exhibit increased intestinal permeability, similar to what exists in cirrhotic patients [105]. This increased permeability resulting from disruptions of the intestinal barrier tight junctions allows for endotoxin translocation into the portal system [106], leading to impaired bile acid metabolism, which in turn promotes systemic inflammation along with alterations in the local microenvironment. Bile acid in the gut can help to maintain physiologic pH levels of the intestinal environment, inhibit the growth of pathogens, and directly interact with the host immune system. Taken together, these roles are vital in maintaining both the local microbial environment and immunologic homeostasis [107].

To characterize the gut microbiota of liver transplant recipients in comparison to healthy individuals, Lai et al. analyzed fecal samples from patients who received liver allografts and uncovered significant alterations in multiple characteristic species, including *Ruminococcus*, *Blautia*, and *Bifidobacterium* [108]. Previous reports demonstrated greater relative abundances of *Enterobacteriaceae* and *Enterococcus* species and a decrease in *Faecalibacterium prausnitzii* and *Bifidobacterium* among liver transplant recipients [48,108,109]. In line with these findings, a recent study by Kato et al. suggests that liver transplant recipients with biopsy-proven rejection have significantly lower microbial diversity, as evaluated by Shannon diversity index, than those without rejection [103]. The authors further reported that in those experiencing acute rejection, the families *Bacteroides*, *Enterobacteriaceae*, *Streptococcaceae*, and *Bifidobacteriaceae* had increased relative abundancies, while patients without rejection had relative increases in *Enterococcaceae*, *Lactobacillaceae*, *Clostridiaceae*, *Ruminococcaceae*, and *Peptostreptococcaceae* [103] (Table 2).

Interestingly, many of these studies revealed that after transplant there is limited recovery of the enteric flora with increased heterogeneity and expansion of eubiotic species, or species that help to maintain a healthy gut microbiome [48,103]. Formal analysis of stable patients six months after transplantation by Bajaj et al. showed mild restoration of microbial diversity and proportional increases of seemingly beneficial *Ruminococcaceae* and *Lachnospiraceae* families and a drop in pathogenic bacteria of the *Enterobacteriaceae* genera, including *Escherichia*, *Salmonella*, and *Shigella* [111]. This local reversion was associated with restoration of the local bile acid cycle and mitigation of the harmful effects of endotoxemia, which taken together, signal a transition to a healthier gut microbiome [111]. However, in another study by the same group, the authors countered that, while there may be some restoration of the microbiome after liver transplantation, dysbiosis with greater proportions of *Proteobacteria* and lower quantities of *Firmicutes* may persist even after six months, potentially resulting in worse outcomes [112]. In accordance with these findings, Lee et al. redemonstrated long-term dysbiosis in liver transplant recipients which was characterized by a decrease in *Faecalibacterium* and elevated levels of harmful *Bacteroides* [113].

Mesenteric lymph nodes play a pivotal role in the development and education of host immune cells and are a heavily influenced by the regional commensal bacteria [114]. The presence of dysbiosis can negatively alter CD4+ T cell subsets and subsequent migration of these modified immune cells can lead to the development of hepatic injury [115] or precipitation of acute rejection. Given the known effects of gut microbial dysbiosis associated with gut barrier dysfunction, increased bacterial translocation can increase antigen presentation in the liver and subsequent simulation of effector T cell differentiation, causing an enhanced alloimmune response, thereby increasing the risk for acute rejection [116,117]. It has been demonstrated that gut microbial dysbiosis can lead to elevated levels of inflammatory cytokines, increasing the risk for acute rejection [118]. Pre-clinical work in rats has shown a decrease in the genus *Faecalibacterium prausnitzii* and *Lactobacillus* accompanied by a rise in *Clostridium bolteae* in animals experiencing acute rejection [119]. If similar effects hold true in the liver, it is possible that an increased presence of *Faecalibacterium prausnitzii* can lead to protective effects against acute rejection [120].

Work by Salimov et al. highlighted a significant difference in the taxa *Protobacteria* and *Enterobacteriaceae* between patients with acute graft rejection and those without [110]. Notably, the authors revealed a significant change in the abundance in the taxon *Candidatus saccharibacteria*. This bacterium is a known epiparasite that can kill host bacteria within certain environments [121]. Thus, *Saccharibacteria* can alter the host’s bacterial physiology by affecting host dynamics or directly altering relative bacterial abundancies through bactericidal effects, which can potentially influence allograft outcomes. Despite these interesting findings, future studies are needed to determine the effects and mechanisms behind the influence of the gut microbiome on acute rejection of liver allografts.

### 5.3. Gut Dysbiosis and Post-Transplant Mortality in Liver Allograft Recipients

In assessing the association between gut microbial dysbiosis and overall survival of transplant recipients, it has been demonstrated that liver transplant recipients with a low Shannon diversity index have a significant increase in post-transplant mortality. Within 3 years of transplant, 77% of recipients identified as having a low Shannon diversity index survived, in comparison to 96% of those with a high diversity index [23]. It was found that with one unit decrease in microbial diversity, the overall mortality risk for transplant recipients increased by 45% [23]. Similarly, a larger Aitchison distance from the general population was associated with a greater likelihood of death among liver transplant recipients. Upon further investigation of specific species associated with increased overall mortality in the liver transplant population, Swarte et al. uncovered that *Atopobium*, *Coprobacillus*, and *Megamonas* were linked with a greater risk of mortality. Conversely, the authors also demonstrated that increased abundancies of *Subdoligranulum* were associated with a significantly decreased risk of mortality. Multiple prior studies have demonstrated a relationship between the Enterococcaceae family and increased risk of mortality in liver disease patients [98] and *Enterococcus* was associated with an increased risk of all-cause mortality after liver transplantation [23].

## 6. The Gut Microbiome and Outcomes After Lung Transplantation

### 6.1. Gut Dysbiosis in Patients with End Stage Lung Disease

As previously demonstrated in patients with ESRD and ESLvD, patients with ESLgD are also characterized by intestinal microflora dysbiosis [23]. In comparison to healthy controls, Zhang et al. found that patients with ESLgD had a significantly lower Shannon diversity index and altered gut microbial composition [122]. The authors also demonstrated that patients with ESLgD were enriched with *Bifidobacterium dentium*, *Bacteroides ovatus*, and *Parabacteroides johnsonii*. Commensal species of *Bifidobacterium adolescentis*, *Akkermansia muciniphila*, and *Agathobaculum butyriciproducens* were present in larger quantities among healthy controls compared to those with ESLgD. In a longitudinal analysis of ESLgD patients up to 24 months from transplant, a greater prevalence of *Escherichia coli*, *C. symbiosum*, and *Anaerotignum lactatifermentans* were also found.

### 6.2. Gut Dysbiosis After Transplantation and Lung Allograft Rejection

Results of clinical and pre-clinical studies have shown influence of gut microbes on lung immunity through multiple different pathways including endotoxins, cytokines, and hormones which work together to comprise the “gut-lung axis” [123,124]. In addition to being on life-long immunosuppressive therapy like liver and kidney transplant recipients, lung transplant recipients often necessitate higher doses of these drugs given the immunogenicity of lungs in conjunction with increased antibiotic requirements due to the greater risk of infection with multidrug-resistant bacteria [125,126]. As a result of the increased use of immunosuppressive medications and antibiotics, lung transplant recipients are notably inclined to develop intestinal dysbiosis, putting lung transplant recipients at risk of worse post-transplant outcomes given the previously described interactions between the gut microbiota and systemic immunity. Interestingly, while the Shannon diversity index was similar between ESLgD and lung transplant recipients, microbial composition varied between the two groups [122]. Overall, in comparison to the healthy population, the diversity of the enteric microbiome among lung transplant recipients was persistently decreased even after 20 years after transplant [122]. The authors of this work also demonstrated higher abundances of *Blautia wexlerae*, *Rothia mucilaginosa*, and *Roseburia intestinalis* and decreased relative abundancies of *A. muciniphila*, *Eubacterium rectale*, and *B. adolescentis*, known SCFA-producing bacteria, with increases in the genera *Clostridium* and *Streptococcus*.

Chronic lung allograft dysfunction (CLAD) is a common and potentially catastrophic complication that impacts about 50% of lung allograft recipients within the first 5 years of lung transplantation [127]. Prior studies have demonstrated the effects of the local pulmonary microflora on the development of acute rejection and graft survival, with greater lower respiratory tract microbial load and decreased diversity being directly linked with increased incidence of rejection and worse survival [128,129]. However, future studies are needed to further investigate the gut microbiome and its effects on lung allograft outcomes.

Pre-clinical work by Wu et al. in murine models has shown that antibiotic pretreatment of lung transplant recipients resulted in decreased severity of rejection [130]. In assessing differences in relative abundances of microbes, it was found that mice who underwent antibiotic pretreatment demonstrated a greater abundance of *Bacteroidetes* and decrease in *Firmicutes* [130]. Notably, a greater quantity of *B. dorei* was negatively correlated with allograft rejection and fibrosis while multiple *Clostridial* species demonstrated a positive correlation [130]. Early investigations into the effects of the enteric microbiome by Zhang et al. did not uncover any noteworthy associations between specific bacteria and CLAD stages, although findings may have been limited by the small sample size [122]. A larger clinical study by Wu et al. (Table 3) found that *Bacteroides uniformis* was significantly reduced in recipients with acute rejection and the authors hypothesized that this microbe may be the main driver for the altered microbial community in this patient population. They also found an enhanced inflammatory state among those experiencing acute rejection which was positively correlated with the increased presence of *Enterococcus* spp. and *Lactococcus* spp. [131]. While these findings are interesting, more studies are needed to characterize the gut microbiome further and pave the way for future therapeutic interventions in lung transplant recipients.

## 7. The Gut Microbiome and Outcomes After Heart Transplantation

### 7.1. Gut Dysbiosis in Patients with Advanced Heart Failure

Similar to observations in other end stage organ disease states, previous studies have demonstrated gut microbial dysbiosis in patients with heart failure [132,133]. A recent study by Spehlmann et al. demonstrated a connection between the degree of intestinal dysbiosis and the severity of HF in a murine model [134]. The authors found that among mice with HF, there was a significant decrease in alpha-diversity, a measure of species diversity within a local area. Key findings in mice experiencing HF included decreased abundancies of *Lachnospiraceae* along with increased abundancies of *Bacteroides*, *Prevotellaceae*, *Alistipes*, *Parasutterella*, *Ruminococcaceae*, and *Ruminococcus*. Studies by both Spehlmann et al. and Leonel et al. highlighted differences in the microbial metabolic profile in those experiencing AHF, specifically surrounding the metabolism of SCFAs. Leonel et al. reported a reduction in butyrate-producing bacteria in this population. Butyrate is a known source of energy for local enterocytes and its absence can impair the integrity of the intestinal barrier and promote local inflammation [135]. In addition to this alteration in the microflora, AHF can also result in intestinal hypoperfusion, further impairing the epithelial border and worsening already reduced microbial richness [136].

In comparison to investigations into the gut microbiome in patients with end-stage kidney or liver disease, that of patients with ESLgD or AHF remains to be further characterized. Investigations into a small group of patients experiencing HF with reduced ejection fraction by Luedde et al. uncovered a notable decrease in relative abundancies of the families *Ruminococcaceae*, *Coriobacteriaceae*, and *Erysipelotrichaceae* [137], while another study demonstrated a relative expansion of *Candida* and other pathogenic bacteria, including *Campylobacter*, *Yersinia enterocolitica*, *Shigella*, and *Salmonella* [138]. These findings were corroborated in larger study conducted by Yuzefpolskaya et al. among patients with heart failure, left ventricular assist devices, and heart transplants, which demonstrated a progressively decreasing level of alpha diversity corresponding with worsening HF class and alterations predominately among the family of *Lachnospiraceae* and *Ruminococcaceae* [133].

### 7.2. Gut Dysbiosis After Transplantation and Cardiac Allograft Rejection

Studies in pre-clinical murine models have demonstrated a relationship between the gut microbiota and cardiac allograft rejection. Investigations by Lei et al. showed that in mice who underwent pretreatment with antibiotics, there was a significant delay in graft rejection, highlighting the overarching interplay between microbiota on allograft rejection [21]. Additional murine studies by Wang et al. have shown that colonization with *Listeria monocytogenes* resulted in the loss of previously developed allograft tolerance and promoted acute rejection in heart transplant recipients [139]. Conversely, transfer of *Bifidobacterium pseudolongum* was associated with improved survival and decreased development of allograft rejection and fibrosis [140,141]. Together, these studies demonstrate the significant connection between the microbiome and allograft tolerance in heart transplant recipients.

Because the heart is not directly colonized by microbiota, interactions between the microbiome and the heart occur indirectly through the recipient’s immune system and bacterial byproducts [139,142]. For example, Troseid et al. uncovered a relationship between the microbiota-dependent metabolite γBB and levels of carnitine with an increased incidence of acute rejection [143]. Further characterization of the gut microbiome of heart transplant recipients was performed by Dela Cruz et al., demonstrating that heart transplant recipients have decreased populations of *Roseburia*, *Ruminococcus*, and *F. prausnitzii*, microbes which are known to produce large quantities of butyrate [144]. While the authors provided some insight into the alternations of the gut microflora in heart transplant recipients, there still is a significant need for additional studies to further characterize the taxa associated with rejection and mortality after heart transplant.

## 8. Limitations

While this review represents one of the most comprehensive overviews of the current associations between the gut microbiome and outcomes of solid organ transplantation, there remain limitations to this work. Although the authors conducted an extensive review of the literature, it is possible that some studies were not captured and thus not included in the manuscript. Additionally, given that capturing robust data on the gut microbiome of solid organ transplant recipients can be a resource-intensive process and requires targeted recruitment, many of the studies mentioned in this review have varied sample sizes and non-uniform control groups, thus drawing definite, causal conclusions regarding the effects of specific bacteria on allograft rejection or mortality may not be possible. Lastly, in conjunction with the previously mentioned point, within the human population, there are a myriad of both inherent and external factors that influence the gut microbiome on an individual level and are subsequently hard to control for within clinical studies. Thus, it is possible that there are other unknown factors at play that may be influencing the uncovered associations between specific microbes and allograft outcomes that are described in the included studies. Overall, these limitations highlight the magnitude of what remains to be discovered in this realm of transplant and the gut microbiome.

## 9. Conclusions and Future Directions

In summary, this review underscores the profound connection between the gut microbiome and outcomes in solid organ transplantation. The highlighted studies demonstrate that gut microbial dysbiosis is intricately linked with allograft rejection, morbidity, and mortality across various types of transplants, including kidney, liver, lung, and heart.

Microbial diversity and specific microbial taxa play critical roles in modulating immune responses, influencing both the success and complications of transplantation. Consequently, the gut microbiome presents a promising target for therapeutic intervention. Approaches such as antibiotic pretreatment, the use of probiotics or prebiotics, and fecal microbiota transplantation could be harnessed to enrich beneficial microbial taxa and eliminate pathogenic ones, potentially leading to improved transplant outcomes. Similarly, profiling of recipient microbiomes could guide personalized immunosuppressive therapies with the potential to improve patient morbidity and mortality.

While the current body of evidence is compelling, it also highlights the need for further research. Future studies should focus on elucidating the specific mechanisms by which the gut microbiome influences immune responses in transplant recipients. Additionally, large-scale longitudinal studies are necessary to validate the microbiome as a reliable biomarker and to assess the efficacy of microbiome-targeted therapies. In summary, the gut microbiome represents both a critical factor in transplant success and a promising frontier for therapeutic innovation. By continuing to explore and understand this complex relationship, we can move closer to improving the lives of solid organ transplant recipients.

## Figures and Tables

**Table 1:** Microbiota associated with graft rejection or mortality in kidney transplant recipients.

Manuscript	Author	Association	Comparison	Study Design	Microbes	Statistical Significance
Human Microbiota Characterization in the Course of Renal Transplantation	Fricke et al. [47]	Rejection	Pretransplant rectal samples compared between patients who experienced later rejection events compared to those without post-transplant adverse events	Prospective cohort	↓ *Anaerotruncus* ↓ *Coprobacillus* ↓ *Coprococcus* ↓ *Peptostreptococcaceae* (unknown member)	*p* < 0.05 * p < 0.05 * p < 0.05 * p < 0.05 *
Gut Microbial Community Structure and Complications Following Kidney Transplantation: A Pilot Study	Lee et al. [77]	Rejection	Fecal specimens from acute rejection recipients to time-matched control samples from the recipients without acute rejection	Prospective cohort	↑ *Lactobacillales* ↓ *Bacteroidales* ↓ *Clostridiales*	*p* = 0.04 *p* = 0.03 *p* = 0.01
Distinct Changes in Gut Microbiota of Patients with Kidney Graft Rejection	Visconti et al. [86]	Rejection	Fecal specimens between transplant recipients who experienced acute rejection vs not	Cross-sectional case-control	↑ *Bacteroides* (acute T-cell mediated rejection) ↑ *Eubacterium* (acute T-cell mediated rejection) ↓ *Alloprevotella* (borderline rejection) ↓ *Faecalibacterium* (chronic T-cell mediated rejection) ↓ *Tannerellaceae* (chronic T-cell mediated rejection) ↑ *Lachnoclostridium* (chronic T-cell mediated rejection) ↑ *Escherichia shigella* (antibody mediated rejection) ↓ *Bacteroides* (antibody mediated rejection)	*p* = 0.02 *p* = 0.03 *p* = 0.04 *p* < 0.001 *p* < 0.001 *p* = 0.003 *p* = 0.002 *p* < 0.001
Gut Microbiota Alterations Associated with Antibody-Mediated Rejection After Kidney Transplantation	Wang et al. [87]	Rejection	Fecal specimens between transplant recipients who experienced antibody-mediated rejection vs not	Case-control	↑ *Erysipelotrichi* ↑ *Lactobacillales* ↑ *Erysipelotrichales* ↑ *Erysipelotrichaceae* ↑ *Bacilli* ↓ *Clostridia* ↓ *Clostridiales* ↓ *Roseburia*	*p* = 0.0068 *p* = 0.0022 *p* = 0.0068 *p* = 0.0068 *p* = 0.0031 *p* = 0.0009 *p* = 0.0009 *p* = 0.0025
Integrative Metagenomic and Metabolic Analyses Reveal the Role of Gut Microbiota in Antibody-Mediated Renal Allograft Rejection	Li et al. [84]	Rejection	Fecal specimens between transplant recipients who experienced antibody-mediated rejection vs not	Case-control	↑ *Klebsiella phage KP8* ↑ *Lactobacillus fermentum* ↑ *Enterococcus phage IME-EFm1* ↑ Streptococcus sp. I-P16 ↓ *Roseburia intestinalis* ↓ *Eubacterium rectale* ↓ *Blautia obeum*	AUC = 0.5411 AUC = 0.6716 AUC = 0.4526 AUC = 0.6316 AUC = 0.6232 AUC = 0.7916 AUC = 0.6779
Unraveling Intestinal Microbial Shifts in ESRD and Kidney Transplantation: Implications for Disease-Related Dysbiosis	Yan et al. [88]	Rejection	Fecal specimens between transplant recipients experiencing borderline rejection vs not	Prospective cohort; Sub-cohort	↑ *Curtobacterium* ↑ *Flexilinea* ↑ *Enorma* ↑ *Coprococcus* ↑ *Muribaculum* ↑ *Planctopirus*	BH adjusted *p* = 0.04 BH adjusted *p* = 0.04 BH adjusted *p* = 0.04 BH adjusted *p* = 0.04 BH adjusted *p* = 0.04 BH adjusted *p* = 0.04

AUC = Area under the curve (receiver operator curve); BH = Benjamini-Hockberg; * No additional information regarding *p*-values provided; ↑ = Increased relative abundance; ↓ = Decreased relative abundance.

**Table 2:** Microbiota associated with graft rejection or mortality in liver transplant recipients.

Manuscript	Author	Association	Comparison	Study Design	Microbes	Statistical Significance
Longitudinal Analysis of the Intestinal Microbiota in Liver Transplantation	Kato et al. [103]	Rejection	Fecal specimens from transplant recipients before transplantation compared to their same specimens during a rejection episode	Prospective cohort study	↑ *Bacteroides* ↑ *Enterobacteriaceae* ↑ *Streptococcaceae* ↑ *Bifidobacteriaceae* ↓ *Clostridiaceae* ↓ *Ruminococcaceae* ↓ *Peptostreptococcaceae*	*p* = 0.4 *p* = 0.02 *p* = 0.02 *p* = 0.03 *p* = 0.02 *p* = 0.03 *p* = 0.0
Gut Microbiota Might Influence the Risk of Rejection After Liver Transplantation	Salimov et al. [110]	Rejection	Fecal specimens between transplant recipients experiencing acute rejection vs not	Prospective cohort study	↑ *Protobacteria* ↑ *Enterobacteriaceae* ↑ *Chloroflexia* ↑ *Chlamydiia* ↑ *Gammaproteobacteria* ↓ *Candidatus Saccharibacteria*	*p* = 0.001 *p* = 0.001 *p* = 0.004 *p* = 0.01 *p* = 0.01 *p* = 0.04
Gut Microbiome Dysbiosis is Associated with Increased Mortality After Solid Organ Transplantation	Swarte et al. [23]	Mortality	Relative abundance of microbe between patients prior to transplant compared to after transplant and associated risk of mortality	Cross-sectional; Longitudinal analysis	↑ *Atopobium* ↑ *Coprobacillus* ↑ *Megamonas* ↑ *Subdoligranulum* *	Adjusted HR: 1.50; 95% CI 1.18–1.92 Adjusted HR: 1.78; 95% CI 1.22–2.60 Adjusted HR: 1.50; 95% CI 1.16–1.94 Adjusted HR: 0.67; 95% CI 0.52–0.87

CI = confidence interval; HR = hazard ratio; * Associated with a decreased risk of mortality; ↑ = Increased relative abundance; ↓ = Decreased relative abundance.

**Table 3:** Microbiota associated with graft rejection or mortality in lung transplant recipients.

Manuscript	Author	Association	Comparison	Study Design	Microbes	Statistical Significance
Intestinal Microbiota Links to Allograft Stability After Lung Transplantation: A Prospective Cohort Study	Wu et al. [131]	Rejection	Fecal specimens between transplant recipients experiencing acute rejection vs. not	Prospective cohort	↓ *Bacteroides uniformis*	*p* = 0.000854175

↓ = Decreased relative abundance.
